# Characterization and Management of Juxtaglomerular Cell Tumor: Analysis of 9 Cases and Literature Review

**DOI:** 10.4274/balkanmedj.galenos.2020.2019.12.79

**Published:** 2020-08-11

**Authors:** Shuangjian Jiang, Yun Yang, Rongpei Wu, Qiyun Yang, Chi Zhang, Yiming Tang, Chengqiang Mo

**Affiliations:** 1 Department of Urology, The First Affiliated Hospital of Sun Yat-Sen University, Guangzhou, China

**Keywords:** Dynamic enhanced computed tomography, juxtaglomerular cell tumor, kidney neoplasm, secondary hypertension

## Abstract

**Aims:**

Juxtaglomerular cell tumor is a rare kidney tumor. This study aimed to report the clinic features of juxtaglomerular cell tumor and our treatment experience.

**Methods:**

The medical records of 9 juxtaglomerular cell tumor patients treated in our hospital from 1997 to 2017 were retrospectively reviewed. Clinical characteristics, immunohistochemical findings, treatments and outcomes were collected.

**Results:**

The mean age of 9 patients was 24±8.1 years (range: 18-37). All cases had symptoms of hypertension, hyperaldosteronism, high plasma renin, high plasma angiotensin II. Four cases had hypokalemia. The renal masses were found by enhanced contrast tomography in all patients. One case received ultrasound-guided ablation and was clinically diagnosed with juxtaglomerular cell tumor. Among the remaining 8 cases, 2 cases received nephrectomy while 6 underwent partial nephrectomy. The 8 cases were pathologically diagnosed with juxtaglomerular cell tumor. Immunohistochemical findings showed that juxtaglomerular cell tumor was positive for vimentin, CD34, and actin but negative for chromogranin A. After treatment, all the patients had normal levels of blood pressure, serum renin activity, potassium, and aldosterone. No patients had tumor progress or metastasis within a median follow-up period of 94 (range: 33-241) months.

**Conclusion:**

Hypertension combined with hyperaldosteronism and hypokalemia secondary to high plasma renin activity are the typical symptoms of juxtaglomerular cell tumor. Partial nephrectomy is an optimal treatment for juxtaglomerular cell tumor.

Juxtaglomerular cell tumor (JGCT), also known as reninoma, is a rare tumor from the juxtaglomerular cell of the kidney. Typical symptoms of JGCT include hypertension, hypokalemia, and renal mass with renin secreting leading to hyperaldosteronism (1). JGCT usually occurs in young adults between 20-30 years (2,3). JGCT was first described by Robertson et al. (4) in 1967. At present, appropriately 100 cases have been reported. Nevertheless, the majority of previous studies are case reports due to its rarity. In this study, we reported the clinical and pathological characteristics of 9 cases of JGCT, as well as the treatment methods and outcomes.

## MATERIALS AND METHODS

### Patients

A total of 9 JGCT patients treated in our hospital from 1997 to 2017 were included. The medical records were retrospectively reviewed. This study was approved by the institutional review board of our hospital. Written informed consent was obtained from each patient.

### Data collection

The data of maximum blood pressure, serum potassium concentration, plasma renin activity, plasma aldosterone concentration, computed tomography (CT), ultrasonography, treatment, hematoxylin and eosin (H&E) staining, immunohistochemistry (IHC), follow-up and prognosis outcomes were collected.

### Treatments

### Ultrasound-guided ablation

All surgeries were performed under local anesthesia combined with intravenous sedation. After anesthesia, a Tru-Core 18 G biopsy needle was first punctured percutaneously to acquire tumor tissue for punch biopsy. Then a mono-pole radio-frequency ablation needle (cool-tip TM Valleylab, Radionics, USA) was punctured at the same point. Under the guidance of the dynamic ultrasound, the needle tip was put into the center of the tumor. The ablation power was controlled to ensure local coagulative necrosis with a temperature above 60°C. When the ultrasonography showed a high-echo gathered around the needle tip, the point was accomplished with coagulative necrosis and then the needle should be put into another place until all the tumor was completely ablated with a high-echo on the ultrasonography. Before the end of the surgery, the power of the ablation needle should be increased to get a temperature of 90-100°C for at least 10 seconds and then pull out the needle to carbonize the needle tract and prevent implantation. After surgery, pressure dressing was implemented to prevent bleeding.

### Nephrectomy and partial nephrectomy

Both nephrectomy and partial nephrectomy were performed through retroperitoneal laparoscopy. After general anesthesia, the patient was placed in a lateral position. Four trocars were used to set up the retroperitoneal space. In the nephrectomy, the whole kidney and the perinephric fat were resected; while in the partial nephrectomy, only the tumor and its surrounding fat were resected. Radical resection was performed in all partial nephrectomy without residual tumor.

### H&E staining and IHC

The surgical tissue was fixed in 10% neutral-buffered formalin and then paraffin-embedded. Paraffin-embedded tissue blocks were sectioned into 3-4 μm sections for H&E staining and IHC. The H&E staining was performed by the routine method. For the IHC, the sections were deparaffinized and rehydrated, and then antigen retrieval was performed by incubating with 10 mM citrate buffer (pH 6.0) and microwaving. The endogenous peroxidase activity was blocked by incubating with 0.5% H_2_O_2_ in 50% methanol for 30 min. The sections were then incubated in 2% BSA for 1 h at RT to block non-specific binding. Then, the sections were incubated overnight at 4°C with the following primary antibodies: anti-Vimentin (V9, Dako, Japan), anti-CD34 (QBEnd/10, Dako), anti-cytokeratin (AE1/AE3; Dako), anti-actin (1A4 clone; Dako), and anti-chromogranin A (A 0430, Dako) antibodies. After incubating with secondary antibody (Nanjing KeyGen Biotech Co., Ltd, Jiangsu, China) at 37°C for 30 min, the sections were stained with diaminobenzidine, counterstained with hematoxylin, dehydrated to xylene through an ethanol series and mounted. Finally, sample sections were observed under a light microscope (Zeiss Axioplan 2, Berlin, Germany).

## RESULTS

### Patients’ demographic and clinical characteristics

A total of 9 JGCT patients (3 females and 6 males) were included for analysis. The demographic and clinical characteristics were shown in Table 1. The mean age at diagnosis was 24±8.1 (range: 18-37) years. All cases had hypertension. The systolic blood pressure of the upper limb was 163.1±16.3 (range: 142-190) mmHg and the diastolic pressure was 108.9±20.1 (range: 70-140) mmHg. Four patients had a paroxysmal headache while 2 cases had paroxysmal dizziness. Four patients with hypokalemia had a mean plasma potassium level of 2.81±0.47 (range: 2.15-3.2) mmol/L. In a supine position, the mean plasma renin activity of the 9 cases was 9183.8±3736.2 (range: 3599-12000) ng/L/h; the mean angiotensin II level was 218.3±225.8 (range: 53.6-800) pg/mL; the mean aldosterone level was 0.622±0.296 (range: 0.33-1.12) nmol/L. The adrenocorticotropic hormone, corticosteroid, and vanilmandelic acid levels were all within their normal ranges.

**Figure 1:**
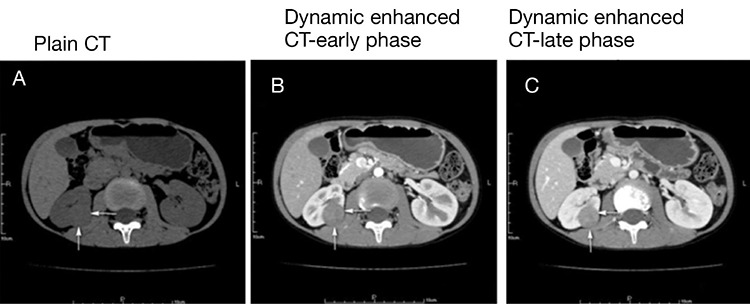
**A-C.** Computed tomography scan of juxtaglomerular cell tumor. Representative images of plain computed tomography scan (A), dynamic enhanced computed tomography scan at the early phase (B) and late phase (C) were shown.

All patients received dynamic enhanced CT, which showed that the mean tumor size was 2.81±1.95 cm (range: 0.9-6.7, Table 1). The plain CT scan revealed that the tumor had low or low to iso-density with a CT value of 26±8 (range: 18-36) HU (Figure 1A). In the dynamic enhanced process, the tumor was stained moderately during the late phase after contrast enhancement with a value of 58±12 (range: 44-82) HU (Figure 1B and 1C). All the patients underwent a renal artery ultrasound and no narrow renal artery was found. Before surgery, all patients received Captopril or/and Aldactone treatment to maintain the blood pressure below 120/80 mmHg.

### Treatments and outcomes

**Figure 2:**
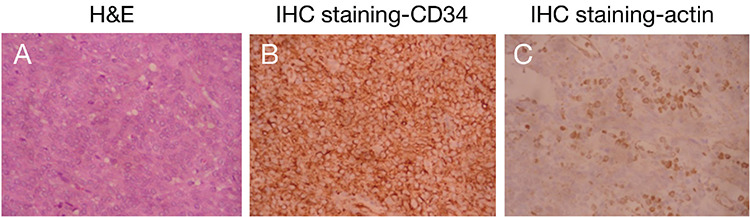
**A-C.** Hematoxylin and eosin staining and immunohistochemistry of juxtaglomerular cell tumor. Representative images of hematoxylin and eosin staining (A), immunohistochemistry staining for CD34 (B), and actin (C) were shown at ×400 magnification.

One patient received ultrasound-guided ablation due to the repulse of laparoscopic or open surgery. After the treatment, the blood pressure, renin level, and aldosterone level all returned to normal. As a result, this case was clinically diagnosed with JGCT. The other eight cases received surgery and were pathologically diagnosed with JGCT. Among them, 2 cases underwent nephrectomy due to the suspicion of a malignant tumor by CT scan. The other 6 cases received nephron-sparing surgery. H&E staining showed that the tumor consisted of solid sheets of closely packed uniform round to polygonal cells with pale to eosinophilic cytoplasm, inconspicuous nucleoli, and indistinct cell border (Figure 2A), indicating the nature of the JGCT tumor was benign in all 8 cases. The immunohistochemical findings demonstrated that the tumor cells were positive for vimentin, CD34 (Figure 2B), and actin (Figure 2C) but negative for chromogranin A (Table 1). All patients had normal blood pressure and plasma potassium level within a week after the treatment. All patients had neither tumor progress nor metastasis during the median follow-up period of 94 months (range: 33-241).

## DISCUSSION

The 9 cases of this report were diagnosed across 20 years. Over the 20 years, there is no considerable change in the diagnostic method for JGCT. The surgical removal technique was mainly open surgery in the 1990s and had gradually switched to endoscopic surgery since 2000. After 2015, robotic therapy was started to be performed for surgical removal of JGCT in our hospital. In the present study, all cases were young adults, which is consistent with previous reports (2,3). However, JGCT has also been reported in 8-year-old child patients (5,6). All cases in our study presented with typical symptoms of JGCT, including hypertension, hyperaldosteronism, high plasma renin, high plasma angiotensin II and hypokalemia. Hypertension in JGCT is caused by high renin and hyperaldosteronism (7). The JGCT patient may suffer from hypertension for many years before the JGCT is diagnosed (8), and the degree of hypertension is not correlated to the tumor size (9). The JGCT patient may also present with symptoms of headache, polyuria, dizziness, and vomiting. These symptoms can be controlled by capton and antisterone before surgical treatment. Dong et al (10). have reported that JGCT can be classified into typical, atypical, and non-functioning types. The patients with typical type can present with typical JGCT symptoms of hypertension, hypokalemia, hyperaldosteronism, and high renin. The atypical type presents with hypertension and renal mass with a normal renin secretion and serum potassium. The patients with non-functioning type have normal blood pressure and potassium levels.

JGCTs were considered to be benign tumors (11), hence nephron-sparing surgery is usually an optimal treatment (12,13,14). However, rare JGCTs with vascular invasion or metastasis were also reported (15). Surgical excision is the curative treatment for JGCT. In this study, 6 out of 9 JGCTs patients received nephron-sparing surgery. With surgical excision, the patients may not need medication (as in the present study) or markedly reduce the medications for blood pressure control (16).

As for imaging examinations, JGCT suspicious patients usually receive an ultrasonography examination firstly. However, dynamic enhanced CT has the highest sensitivity of diagnosis for JGCT (17). In this study, plain CT scan found low or low to iso-density and the dynamic enhanced CT revealed late enhanced stain during enhanced phase with a lower density as compared with the enhanced renal cortex around the tumor. Tanabe A et al. also reported high-density renal mass in the plain CT scan for JGCT (17). For magnetic resonance imaging (MRI), Kang et al. (18) report that iso-intensity or mild hyperintensity on T2WI, a lower ADC value (heterogeneous hyperintensity) on DWI, and a degree of enhancement <200% in the corticomedullary phase were the major MRI findings for JGCTs. Renal angiography can identify whether the hyperreninemia is caused by renal artery stenosis. However, the dynamic CT can also identify renal artery stenosis in a non-invasive way, so we did not perform renal angiography.

In this study, immunohistochemical findings showed that JGCT s were positive for vimentin, CD34, and actin but negative for chromogranin A. Some patients were positive for cytokeratin while the others were not. These findings are in line with previous studies (19,20,21). Martin et al. (19) also reported that the JGCTs are negative for cytokeratin, chromogranin, synaptophysin, HMB-45, S-100, c-kit, CD31, factor VIII, or desmin.

Several limitations of this study should be pointed out. First, this was a case series report with small sample size. Besides, we did not perform genetic analysis on these patients. Moreover, one case receiving ablation without pathology results was clinically diagnosed with reninoma. In the future, a well-designed large prospective clinical trial should be conducted to validate the findings of this study.

In conclusion, JGCT is a rare benign tumor which typically causes hypertension, hyperaldosteronism, hyper-angiotensin, high serum renin, and hypokalemia in young adults of 20-30,s. Dynamic enhanced CT is useful for the positioning and diagnosis of JGCT. Pathological diagnosis of JGCT is defined as IHC staining showing positive for vimentin, CD34, and actin, but negative for chromogranin A. Nephron-sparing surgery is recommended for the treatment. More cases are needed for further study of JGCT.

## Figures and Tables

**Table 1:**
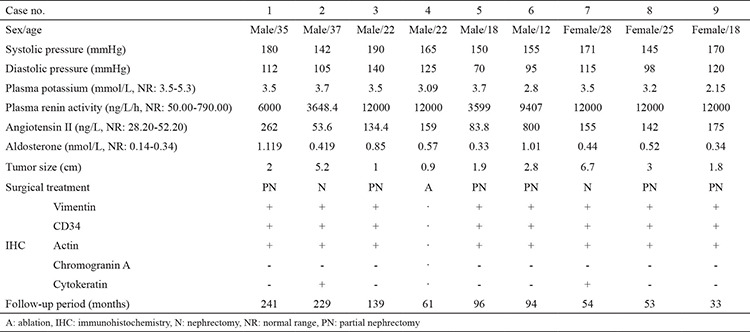
Demographic and clinical characteristics of the patients
